# Shaped 3D microcarriers for adherent cell culture and analysis

**DOI:** 10.1038/s41378-018-0020-7

**Published:** 2018-08-13

**Authors:** Chueh-Yu Wu, Daniel Stoecklein, Aditya Kommajosula, Jonathan Lin, Keegan Owsley, Baskar Ganapathysubramanian, Dino Di Carlo

**Affiliations:** 1 Department of Bioengineering, University of California, Los Angeles, CA USA; 2 Department of Mechanical and Aerospace Engineering, University of California, Los Angeles, CA USA; 3 California NanoSystems Institute, University of California, Los Angeles, CA USA; 4 Jonsson Comprehensive Cancer Center, University of California, Los Angeles, CA USA; 5 Department of Mechanical Engineering, Iowa State University, Ames, IA USA

## Abstract

Standard tissue culture of adherent cells is known to poorly replicate physiology and often entails suspending cells in solution for analysis and sorting, which modulates protein expression and eliminates intercellular connections. To allow adherent culture and processing in flow, we present 3D-shaped hydrogel cell microcarriers, which are designed with a recessed nook in a first dimension to provide a tunable shear-stress shelter for cell growth, and a dumbbell shape in an orthogonal direction to allow for self-alignment in a confined flow, important for processing in flow and imaging flow cytometry. We designed a method to rapidly design, using the genetic algorithm, and manufacture the microcarriers at scale using a transient liquid molding optofluidic approach. The ability to precisely engineer the microcarriers solves fundamental challenges with shear-stress-induced cell damage during liquid-handling, and is poised to enable adherent cell culture, in-flow analysis, and sorting in a single format.

## Adherent cells: microcarriers for flow cytometry

A new microcarrier for adherent cells is demonstrated which allows for accurate flow cytometry and high-speed imaging without risk of flow-induced damage. Microcarriers are attractive for accelerated cell culture, passaging and analysis, but they must be designed to promote cell growth and analysis without flow-induced cell damage. A team led by Dino Di Carlo at University of California, Los Angeles now report a 3D-shaped microparticle that features a region of extracellular matrix for cell adhesion and culture physically protected from shear flow. Key to the design is the intersection of two 2D patterns, leading to a shape which can align with flow inside the channel during cytometry, and also provides a cut-away region to protect cells during culture. These microcarriers may facilitate high-speed adherent cell screening for applications such as drug discovery.

## Introduction

Traditional processes of tissue culture of adherent cells make use of cell growth on flat and rigid polymer petri dishes, flasks, and well plates. Subsequent cell analysis involves scanning the culture surface with microscopy, or bringing cells into suspension with enzymatic or physical treatments followed by flow cytometry to analyze and select sub-populations. This paradigm of cell culture, single-cell enzymatic suspension, and passaging is especially challenging for growth of terminally differentiated cell populations from pluripotent or multipotent precursors^1^. For example, the isolation of retinal pigmented epithelial cells derived from induced pluripotent stem cells cannot be accomplished using standard approaches, but instead requires growth on surfaces followed by manual selection and scraping of pigmented clusters of cells for expansion.

**Fig. 1:**
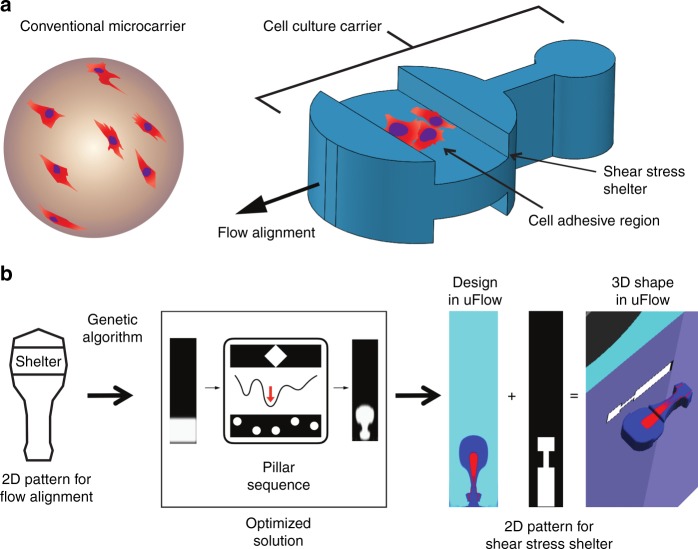
The design of the 3D microcarriers. **a** A conventional spherical microcarrier (left-hand side) and the novel microcarrier (right-hand side) with integrated functionalities achieved by 3D shaping: localized cell culture, shear-stress shelter, and flow alignment. **b** Design flow chart for optical transient liquid molding. To generate a dumbbell shape using inertial flow engineering, a genetic algorithm is executed to optimize the design parameters, including the inlet pattern and pillar sequence. The optimized device design shows the actual pillar sequence with a compressed scale for inter-pillar spacing in order to better view the design. uFlow is used to demonstrate the final dumbbell shape with the cell-adhesive region (red) and 3D shape of the microcarrier when cross-linking through a mask defining the orthogonal notched shelter design

Particle-based cell culture, whereby adherent cells grow and are analyzed on engineered microparticles or microcarriers, can serve as a new paradigm to accelerate culture, passaging, and analysis, without exposing cells to harsh environments^2,3^. Spherical microcarriers, shown on the left-hand side of Fig. 1a, provide a large surface area that enables scaled-up production of anchorage-dependent cells^4–6^. However, it is challenging to sort or further process current spherical microcarriers for several reasons. (1) Cells attached on the sphere are exposed directly to surrounding flows and surfaces, (2) cells growing across the entire curved surface of the sphere are located at different optical focus planes, and (3) the rotation of a sphere makes the locations of the cells change dynamically over time. Additional features can expand the capabilities of these microcarriers, for example, photonic crystal encoding enables evaluation of growth on multiple biomaterials simultaneously^7^. In the past decade, new methods for fabricating microparticles with non-spherical shapes has yielded more advanced functionalities for cell culture, manipulation, and analysis, allowing for more refined exploration of cellular biology using engineering approaches. Sensitive stem cells can be cultured and investigated at the single-cell level using magnetic micro-rafts^8^. Octopus-shaped microparticles provide a new cell-capture strategy for characterizing circulating tumor cells^9^. Interlocking two-dimensional (2D) extruded microparticles with cells embedded allow self-assembly to generate a spatial distribution of various cell types, which is promising for applications in tissue engineering^10^. However, the current capabilities of microparticles have been limited by the ability to engineer the shape and functionality of microparticles in all three dimensions.

To achieve adherent cellular analysis in a precise and high-throughput manner, there is a need to develop engineered microcarriers that can enable growth but also integrate with the advantages of imaging flow cytometry: gathering comprehensive information and detecting signals at high speed simultaneously. The carrier should possess three integrated capabilities: (i) allow cell adhesion and growth, (ii) protect cells from shear stress intrinsic to pipetting and flow-through single-point and imaging flow instruments, and (iii) enable alignment in a microchannel flow cell to achieve uniform velocities necessary for accurate imaging flow cytometry readout^11^. Cell culture should be possible in a protected area while the shape of the carrier self-guides it toward a constant lateral location in channel flows, such that adherent cells can pass through a fixed imaging field of view at a uniform velocity for continuous detection. Through this process, cultured cells should be protected from interaction with flow cell walls or high shear stress from flow fields around the carrier.

In this work, we design, fabricate, and demonstrate the analysis of three-dimensional (3D)-shaped microparticles that act as microcarriers for cell culture and analysis. The microcarrier is designed to have a localized area of extracellular matrix for cell adhesion and culture in a shear-protected nook, which allows high-speed transportation and imaging of adherent cells in channel flows with minimized shear stress. The microcarrier is shaped by the orthogonal intersection of two 2D patterns. One pattern allows for the alignment of the microcarrier in flow and provides an isolated cell culture region, while the other pattern has a depressed region to serve as a shear-stress shelter with a width designed to match the dimensions of a microchannel and allow for “quasi-2D” alignment in flow (Fig. 1a). To focus the particles in flow, we design the particle along one dimension with an asymmetric dumbbell shape that has been shown to self-align in Stokes flows^12^. A cell-adhesive region is designed to be encapsulated inside the boundary of this shape and surrounded by a region of polymer with low binding affinity. In the orthogonal direction, the shear-sheltering region consists of notches on both sides of a rectangular shape with a height designed to be 90% of the height of the microchannel flow cell. The lateral position and length of the notches are aligned with the cell-adhesive region patterned in orthogonal projection (Fig. 1a).

## Materials and methods

### Microcarrier fabrication

We make use of a novel microparticle manufacturing approach called optical transient liquid molding (OTLM), which is capable of producing new classes of complex microparticles with software-designed 3D shape and functionality^13^. OTLM generates microparticles by illuminating patterned ultraviolet (UV) light onto a target flow stream of polymer precursor. The flow stream of precursor materials is shaped in a pre-engineered cross-sectional pattern in a microchannel using fluid-structure interactions, which occur at a flow rate (or Reynolds number, *Re*) higher than conventional microfluidic systems are operated. The advection of flow in the cross-section due to these finite inertia interactions with obstacles is called inertial flow deformation. Inertial flow deformation around micropillars is used to sculpt the cross-sectional pattern of these co-flows (streams of photo-crosslinkable monomer with and without photoinitiator) as a single phase at Reynolds (*Re*) number between 1 and 100^14^. Irreversibility at these *Re* numbers (inertial regime) breaks aft-fore symmetry, generating net secondary flows in the cross-sectional plane that depend on the location and size of the micropillars, flow conditions (*Re*), and channel aspect ratio. By eliminating the hydrodynamic coupling between micropillars, we can engineer and predict the cross-sectional pattern of the target flow in a rapid manner using software, uFlow^15^, developed in our labs, which we have made freely available to the public (http://biomicrofluidics.com/software.php). We have demonstrated that fundamental and complex patterns can be created by arranging the size and sequence of micropillars^14,16^. To produce complex microparticles, we developed an automated fluidic and optical system, OTLM, where upstream/downstream pressure and optical shutters are controlled using LabVIEW. Once the designed cross-sectional pattern, created by a pillar sequence, is developed completely in the flow, we impede the flow by equalizing the upstream and downstream pressure and then apply UV illumination through a mask to photocrosslink microparticles in the microchannel. OTLM generates microparticles with levels of asymmetry previously unachievable using flow lithography techniques, with shapes formed by the intersection of two extruded 2D patterns. The approach also allows hybrid particles with multiple functionalities based on differences in content of the co-flowing streams, including biotinylated surfaces and shape-dependent magnetic properties on an individual microparticle^13,17^.

### Genetic algorithm to solve the inverse problem

We design an asymmetric dumbbell shape by using the genetic algorithm to solve the inverse problem, which identifies positions of pillars within the microchannel that satisfy the constraints of our desired shape without any user-based iterations. Previous work using OTLM and uFlow required the user to experiment with a variety of micropillar configurations in order to arrive at a desired cross-sectional shape, a process that could be tedious or difficult to achieve given the extremely large design space ((4 × 8)^10^ = 1.13 × 10^15^ possible configurations for a 10-pillar sequence). We had a choice to design either of the two orthogonal shapes, an asymmetric dumbbell or a notched rectangle, with inertial flow deformation. Given our previous experience that smoothly curved shapes require fewer pillars to achieve, we chose to design the asymmetric dumbbell in the flow direction. To achieve the design without manual iteration, we applied a genetic algorithm approach to reach an optimized initial flow condition and micropillar sequence. The genetic algorithm has been shown to be a useful method for designing inertial flow sculpting devices that produce a desired fluid flow shape^18,19^. Briefly, the genetic algorithm optimizes a population of randomly generated designs by mimicking the process of natural evolution, with random mutations and fitness-based selection evolving the population toward a more optimal design based on a user-specified objective function. We used the freely available software “FlowSculpt” (www.flowsculpt.org), which outputs a device design as a set of volumetric flow rate ratios for streams with and without photoinitiator at the inlet (called the inlet flow pattern) and the pillar sequence to achieve the desired outlet pattern. Because we did not require the sculpted flow shape to be at a particular location in the channel cross-section, the objective function used was a translation invariant image correlation function. This objective function could optimize device design for output flow shapes similar to that defined in Fig. 1b, regardless of their lateral location in the channel (see the Methods section for more detail). After FlowSculpt generated several sets of solutions, we exported the solutions into uFlow, and designed the volumetric flow rate of a sub-stream that included biotin, which we used for the cell-adhesive region in the final particle. Finally, we designed the optical mask for the orthogonal projection of the microcarrier that included a notched rectangle to achieve the shear-stress-shelter pattern. Here, we set the length of the rectangle to be the same as the asymmetric dumbbell, with the notches located at the same lateral position of the cell-adhesive region. The figure on the right-hand side of Fig. 1b demonstrates the 3D shape of the microcarrier for the final design predicted by uFlow.

### Expanding fabrication throughput for cell culture

**Fig. 2:**
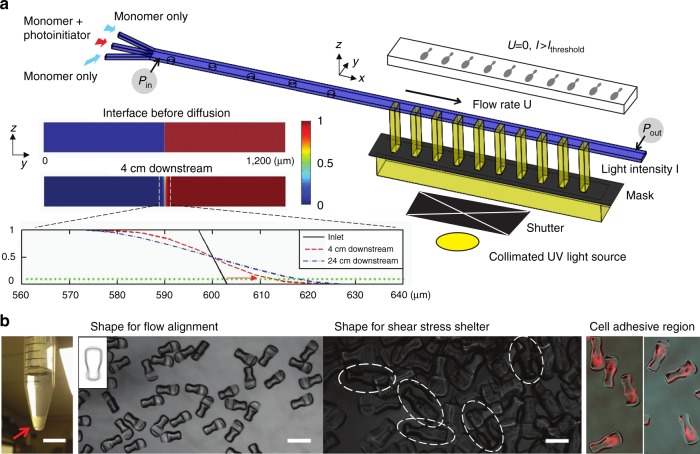
Schematics, simulation, and results for high-throughput OTLM. **a** The fabrication system includes a microfluidic channel with a longer length downstream, flow handling subsystem (for pumping and stopping by altering *P*_in_ and *P*_out_), and UV illumination through a linear array. In the inset on the left-hand side, a convection-diffusion simulation result (only the cross-section above the symmetrical plane of the channel) shows that the diffusion length in the lateral direction of the middle interface along the microchannel at high *Pe* is less than 10% the dimension of fabricated microcarriers, so the dimensional error in a linear array (after stopping flow) is predicted to be negligible. **b** Images of fabricated microcarriers accumulated in the bottom of a 15 mL centrifuge tube are shown on the left (scale bar = 1 cm). Second from the left the microcarrier’s flow alignment dumbbell shape (400 µm scale bar) is shown with an inset showing 135 overlapped outlines. An orthogonal view of the shelter (scale bar = 200 µm), and fluorescently-labeled cell-adhesive regions are shown from on the right-hand side (please view in color)

To generate sufficient number of 3D-shaped microcarriers for repeated biological experiments, we developed a new version of OTLM that achieved a two order of magnitude increase in production rate compared to previous demonstrations (Fig. 2a). In the fabrication system, diluted poly (ethylene glycol) diacrylate (PEGDA, Mn ~575, 40% in DPBS) and 2,2-dimethoxy-2-phenylacetophenone were used for the monomer and photoinitiator in the co-flow, respectively. Biotin-PEG-acrylate was synthesized as an additive to form the cell-adhesive region in the target microcarrier. Its concentration was determined to be 1.5 mg/mL in the diluted PEGDA monomer for the preliminary cell experiments because cell morphology was found to be rounded instead of naturally elongated for lower concentrations. We took advantage of the high Peclet number (*Pe* > 10^6^) of our flow that allowed for the fully developed sculpted flow to maintain its shape for a long channel length downstream. This allowed us to significantly enhance the fabrication rate by synthesizing 100 microcarriers per exposure, instead of one, in an elongated straight microchannel downstream of the flow sculpting region (Fig. 2a). The novel fabrication system required a longer microfluidic chip (fabricated using 6-inch wafer-based soft lithography), as well as a collimated UV light source with a large exposure area to expose the sample through an optical mask in direct contact with the channel with hundreds of patterns in a linear array.

### Imaging and analyzing the microcarriers flowing through a straight microchannel

We utilized microscopy with a high-speed camera to record the images of the microcarriers flowing through a straight microchannel at the inlet and 4 cm downstream. We pumped microcarrier-laden suspension manually and continuously using a syringe pump, respectively, for the case with various *Re* (*Re* ranges from ~6 to ~280) and constant *Re* (*Re* ~20). We first withdraw the microcarriers into a long tubing to avoid settling and then pumped the liquid with a speed high enough that the flow can flush the microcarriers through the narrow region at the inlet or outlet due to their flexibility. Matlab code allowed us to extract the boundary of each captured microcarrier, and calculate its lateral location, orientation, and velocity. The lateral location was determined by the mass center of the boundary, while the orientation was calculated by searching for the angle of rotation with respect to the mass center to reach a minimal aspect ratio. For particle velocity, we calculated the distance of a microcarrier between two sequential frames and multiplied it by the frame rate of the video.

### Cell adhesion on the microcarriers

To demonstrate cell binding on the 3D microcarriers, followed by cell culture, and high-speed imaging of cells on flowing microcarriers, we chose the MDA-MB-231-GFP breast cancer line. We sequentially incubated biotin-modified microcarriers with 1 mg/mL streptavidin and then ~1 mg/mL biotinylated collagen I overnight to saturate the binding sites. The biotinylated collagen was synthesized by mixing solutions of biotin-NHS and collagen overnight, and the non-reacted biotin-NHS was removed using standard dialysis. After each incubation, we rinsed carriers using DPBS solution (10^−3^ M pluronic) with a volume 100 times larger than the solution volume of the microcarriers. We then used DMEM solution (10^−3^ M pluronic) to rinse microcarriers, transferring them into medium solution. A total of 250–300 microcarriers were first settled down on the bottom of an ultra low attachment well (Corning Inc., Corning ultra low attachment surface culture dish, 96-well plate), and then 100 µL of medium solution with suspended cells at a concentration of 10^4^ to 10^5^/mL was dispensed. Accordingly the total number of cells ranged from 1000 to 10,000, which was chosen because we found that the opportunity for cells to interact with the microcarriers was low if the absolute number of cells per well was below 1000. After overnight incubation, we took images of microcarriers, showing that cells resided and spread out on the surfaces of the microcarriers. More data on the cell capture and growth on the microcarriers are shown in the supplementary materials (Fig. S3).

### Cell protection by the design of the shear-stress shelter

General cell manipulation using the 3D microcarriers, including cell culture and high-speed imaging, requires processes of liquid pipetting and flowing of particles in a microchannel. We numerically modeled the shear stress acting on the surface within the shelter for the highest shear-stress step of imaging in a microchannel, in which the microcarrier flows through the rectangular microchannel with a constant flow speed. Calculated shear-stress values were significantly lower compared to values reported for cell survival in the literature (Figs. S1 and S2). In addition, we investigated cell viability following pipetting and the total process of flow cytometric imaging, which provided a practical confirmation of the protective effect of the shelter (Figs. S3 and S4). We also analyzed the cell occupancy on the area of the microcarrier without protection before and after general pipetting. We produced microcarriers with collagen on all surfaces, seeded, and incubated with cells to encourage growth randomly on the surfaces. We then pipetted the microcarrier-laden medium up and down using a 100 µL pipette tip, suspended the microcarriers in bulk medium, settled them back to the well, where we imaged the cells on the microcarriers. We counted the microcarriers with cells inside and outside the shear shelters before and after pipetting/washing and divided it over the total number of microcarriers. To simplify the discussion, we excluded the surface area close to the side surface (with the waists) and only searched for cells residing on the top and bottom flat surfaces of the microcarrier, as the area enclosed by dash lines in Fig. 4a.

### Bright-field and fluorescent imaging of the microcarriers with adhered cells in flow

We pumped the medium with cell-loaded microcarriers into a straight microchannel flow cell to image the adhesive cells on the self-aligned microcarriers. In order to prevent expansion of the hydrogel particles caused by temperature changes during incubation and enable better matching of dimensions to the microchannel and flow alignment, we equilibrated cell-loaded microcarriers at room temperature for 15 min. We then suspended the microcarriers with cells in 500 µL of medium, withdrew the solution into tubing with 1.6 mm inner diameter and 1 m length (to prevent particle settling and loss in the syringe), plugged the tubing into the inlet of the straight imaging microchannel, and pumped the suspension with a volume flow rate of 500 µL/min (channel *Re* ~20). For bright-field imaging, we recorded videos 4 cm downstream in the microchannel using a high-speed camera (Phantom V711, Vision Research) and mercury light source with a short exposure time. For in-situ fluorescent imaging, we adapted the microchannel flow cell to the fluorescence imaging using radiofrequency-tagged emission (FIRE) optical system, which simultaneously excites fluorescence at different spatial locations along a line with different radiofrequency amplitude modulated waves and rapidly recovers the fluorescent images within the line using short-time Fourier Transform^11^. We applied an 80 µm wide excitation line to the center of the microchannel. Each detection event was triggered by a side scatter signature associated with one microcarrier to avoid sensing suspended cells or debris. The microcarriers without cells were first introduced to flow through the line and the speed was calibrated until the correct aspect ratio of the imaged microcarriers was reproduced. Then, the microcarriers with adherent cells on all surfaces were pumped into the microchannel for detection. CellProfiler and Matlab were used for post-processing of the images cropped from the shelter region.

## Results

### High-throughput fabrication of microcarriers with predictable quality

Using the new massively parallel exposure process, we determined that microcarrier dimensions and patterns were largely uniform. Following 1 h of fabrication, we were able to produce over 30,000 microparticles, an amount of microcarriers large enough such that it was visible at the macro scale in a conical tube (Fig. 2b). Images of the microcarriers qualitatively indicate that the orthogonal flow alignment and shear-stress-shelter patterns match the predicted design. The uniformity of the flow-alignment pattern of each microcarrier, which is most dependent on diffusion of photoinitiator downstream, was quantitatively studied using image traces of the boundaries. The averaged deviation of the distance between the boundaries and mass centers is ~12 µm, which is negligible when compared to the dimension of the dumbbell shape. Lastly, we verified that the biotinylation of the cell-adhesive region was uniform by incubating the microcarriers with fluorescently-labeled streptavidin. The red pattern on every microcarrier occupied the same region of the particle (averaged correlation of each pattern and averaged pattern >0.8) in agreement with our design in Fig. 1b.

We also investigated the dimensional error in fabrication caused by diffusion of photoinitiator laterally into the monomer stream using simulations in the inset of Fig. 1a. This diffusion has the potential to expand the sculpted flow shape in downstream regions of the flow when polymerizing a linear array of particles, where the diffusion time is longer. Finite element simulations of the coupled fluid flow and convection-diffusion phenomena identified that for our operating conditions (*Pe* = 2.88 × 10^6^, *Pe* = *UW*/*D*), the central interface along a cross-section of the co-flow shifts ~10 µm after 4 cm of downstream flow, for an average downstream velocity, *U* of 0.24 m/s, channel width, *W* of 1200 µm, and diffusivity of the photoinitiator, *D* of ~10^−6^ cm^2^/s. When lengthening the channel further from 4 cm to 24 cm downstream, only an additional 5 µm expansion in the photoinitiator stream width was observed. Considering microcarriers with dimensions of 200 µm, the predicted dimensional error at these two locations are 5% and 7.5%, respectively, indicating the dimensional variation of microcarriers fabricated along the sculpted flow for significant distances downstream would be negligible. Supported by the simulation results, we enlarged the length of the region downstream of the sculpted flow to include a straight channel with a length of an additional ~4 cm, which required modifications to the optical exposure system. Given the simulation results for 24 cm channel lengths, we expect further increases in throughput—up to another order of magnitude—would be possible by further extending the length of the channel.

### Self-alignment of microcarriers in channel flows

**Fig. 3:**
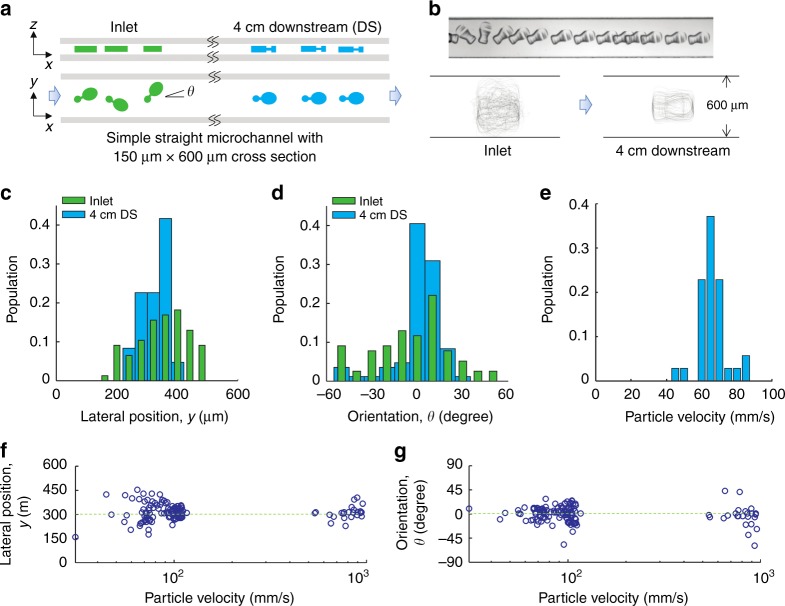
Flow behavior of asymmetric dumbbell microcarriers. **a** Schematic of the top and side views of the microcarriers flowing from the inlet to a location 4 cm downstream in a straight microchannel where particles focus and align. **b** Time-lapsed image (top inset) of a microcarrier self-aligning at low *Re* in a channel with 600 µm width at a position close to the inlet and an overlay of boundaries (bottom insets) of ~50 microcarriers at the inlet and 4 cm downstream at *Re* ~20. **c**–**e** Lateral positions, orientations, and velocity of the microcarriers, quantitatively showing the behavior of self-alignment and a more uniform velocity distribution at *Re* ~20. **f**, **g** Lateral positions and orientations (referring to *y* and *θ* in (a)) of microcarriers flowing and focusing at different velocities, quantitatively showing the behavior of self-alignment over a wide range of *Re*

An important aspect of the microcarrier design is to allow alignment in a microchannel flow cell for flow and imaging cytometry applications that rely on uniform velocity and a defined optical interrogation location. We evaluated the performance of the asymmetric dumbbell flow-alignment design with fabricated microcarriers. We pumped microcarrier-laden solution into a long straight microchannel and observed focusing and alignment using a high-speed camera under various flow conditions. The microchannel had dimensions of 600 µm width by 150 µm height to match the height of the fabricated microarriers. In Fig. 3b, a time-lapse image shows that microcarriers will self-align at low flow rates, rotating until reaching a horizontal configuration with the flow, with the larger lobe of the dumbbell facing downstream. For higher flow rates useful for higher-throughput cytometry (channel *Re* ~20), we continued to observe rotational and positional alignment within the channel at the inlet and 4 cm downstream. The traced boundaries of the particles at the inlet spanned across the whole channel width, while 4 cm downstream the traces converged into the flow-alignment pattern at the center of the channel with the large lobe facing downstream. These results support that the designed pattern enables self-alignment by hydrodynamically interacting with the channel flow. The lateral locations of the centers of mass and orientations of the imaged microcarriers also confirm these results quantitatively (Fig. 3c, d). Compared with wide distributions at the inlet (green), the distributions became significantly tighter downstream (blue) around the center location of the channel and with zero degree alignment with the large lobe facing downstream. Imaging flow cytometry systems, i.e. FIRE and Amnis ImageStream^11,20^, benefit from targets flowing at uniform velocity, which can be aided by particle alignment. We calculated the velocity of microcarriers and as can be seen in Fig. 3e, the velocity distribution was also relatively narrow with a peak velocity of ~65 mm/s, which is promising for future integration. In addition, for generally understanding the flow behavior of the asymmetric dumbbell shape, we investigated the alignment quality as a function of the flow speed of the microcarriers. Each point in Fig. 3f, g shows a lateral position and tilting angle respectively of a microcarrier with different flow velocity imaged in the microchannel. The steady locations and orientations were concentrated respectively around 300 µm and zero degrees, demonstrating that the microcarriers were aligned in the middle of the channel without a tilting angle across different speeds. These data represent the robustness of the self-alignment of the microcarriers in the channel flow across flow rates (~20–1000 mm/s). Therefore, under real-world operation, fluctuations of the flow rate would not be expected to yield a significant effect on performing imaging in the fixed position of the detector, which is a major advantage of self-alignment.

### Shear-protected growth of cells on microcarriers

**Fig. 4:**
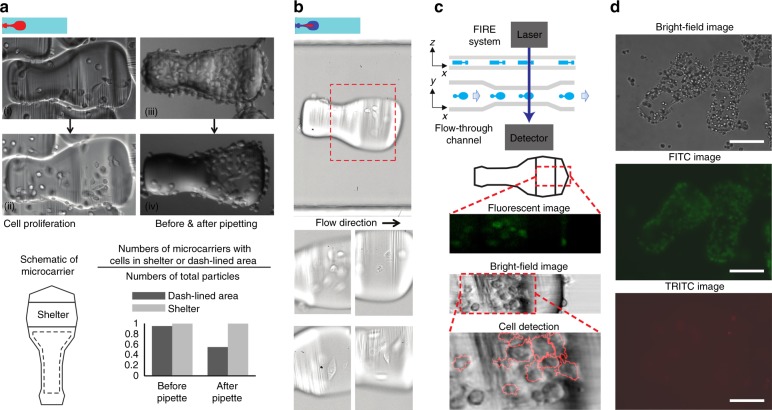
Cell adhesion, proliferation, protection, and analysis using 3D microcarriers. The carriers were biotinylated across all surfaces in (**a**) and partially on the designed region in (**b**) as shown in red color in the top insets. **a** Cell attachment and growth in the shelter after incubation with 266 microcarriers and ~10,000 suspended cells (i, ii), and cell protection provided by the shear-stress shelter during pipetting and liquid-handling (iii, iv). **b** High-speed imaging of the adherent cells on the microcarriers flowing through the straight microchannel with 600 µm width. **c** Schematic and results of integration of the microchannel flow cell with FIRE imaging system, showing high-speed fluorescent imaging of adherent cells on the microcarriers. Green fluorescence is from calcein stained cells. Viable cells are masked based on fluorescence intensity and overlaid on the bright-field image. **d** Viable adherent cells continue to grow in the shear-stress shelter after the flow-through experiment. FITC image shows calcein fluorescence (live stain), TRITC shows propidium iodide (dead stain). The scale bar is 200 µm

To study cell culture on microcarriers and evaluate whether there was a differential survival advantage for cells adhered in the nook area of the microcarrier, we created microcarriers with a collagen coating over the entire carrier. Following incubation of MDA-MB-231-GFP cells with the collagen patterned microcarriers, we observed cell attachment and growth over several days (Fig. 4a, (i)). Cells remained viable during this time period and proliferated (Fig. 4a, (ii)). After incubation of several days, the cells can reach confluency over the entire microcarrier (Fig. 4a, (iii)). We observed that standard processes of pipetting and liquid-handling dislodged cells from most regions of the microcarriers. However, cells were differentially enriched in the area with low shear stress, which includes the shelter and side surface (Fig. 4a, (iv)). Simulation results (Figs. S1 and S2) predict that these are the low stress regions, as designed. The cells in these regions also remained viable. While the design of the shear-stress shelter functioned as expected, the waist of the dumbbell shape also was observed to provide a sheltering effect and enhanced cell adhesion.

We next demonstrated adherent-cell bright-field and fluorescent imaging flow cytometry by guiding the microcarriers with cells through a microfluidic flow cell aligned with two types of optical sensors. Microcarriers flowing through the microchannel flow cell were imaged using a high-speed camera and demonstrated that flow alignment was independent of cell attachment. In these experiments, the patterned adhesive region was included to encourage growth only in the protected shelter of the carrier. The images in Fig. 4b show that MDA-MB-231-GFP cells can be imaged in a high-speed flow with clear morphology. Adherent cells were rapidly transported in the straight microchannel and occupied the same focal plane within a narrow imaging window of ~300 µm using microcarriers. We also utilized fluorescence imaging using radiofrequency-tagged emission (FIRE)^11^ to simultaneously generate bright-field and fluorescent images of adherent cells traveling through a narrow microchannel (300 µm in width), shown in Fig. 4c. The boundaries of imaged cells were detected using CellProfiler (cellprofiler.org), and the result agrees with the bright-field image shown in the overlapping picture on the bottom of Fig. 4c. We demonstrated that the microcarrier system can be integrated with modern imaging flow cytometry to enable high-speed fluorescent detection of adherent cells (Fig. S4). Moreover, to investigate cell viability, which would be important for future sorting of adherent cells on carriers, we collected cell-loaded microcarriers after imaging experiments and continued culture for 2 days. Cells adhered in the sheltering region remained viable by live-dead stain and continued to proliferate (Fig. 4d, Fig. S4b). These data suggest that physical interactions with the flow channel walls and fluid shear stress induced by the flow was lower than the critical threshold causing cell death for cells adhered in the shear-sheltering region.

## Discussion and conclusion

We demonstrated a novel type of 3D-shaped microcarrier allowing particle-based cell culture and high speed imaging of adherent cells in flow, while reducing the fluid-induced forces acting on cells. Taking advantage of the high Peclet number of the sculpted precursor flow, we expanded the manufacturing rate of OTLM to produce microcarriers at two orders of magnitude higher rates and with uniform properties. The microcarriers were shown to automatically self-align in channel flow over a wide range of *Re*, enabling higher resolution high-speed imaging. Adherent cells were shown to remain viable and proliferate even following pipetting and flow through a cytometry flow cell when inside the designed shear-stress shelter of the microcarrier. In the short term, these precision 3D hydrogel microcarriers can be combined with advanced optical analysis, for example, FIRE and Amnis ImageStream^11,20^, for particle-based high content screening, which can be applied to accelerate drug discovery in the pharmaceutical industry. In the long term, we expect these carriers can be combined with image-based analysis (and sorting) of adherent cells to isolate specific rare populations of cells, or sort based on adherent cell or microtissue shape and morphology for the first time. This is a crucial step forward toward automated sorting of differentiated cells or induced pluripotent cell colonies for regenerative medicine.

## Electronic supplementary material

Supplemental Material File #1
