# Supplementary material for: Shaped 3D microcarriers for adherent cell culture and analysis
Source: Microsyst Nanoeng. 2018 Aug 13;4:21. doi: 10.1038/s41378-018-0020-7 (PMC6220171; doi:10.1038/s41378-018-0020-7)
Supplement: Supplementary file 1 — Supplemental Material File #1 [file 41378_2018_20_MOESM1_ESM.pdf]

Supporting Information

SHAPED 3D MICROCARRIERS FOR ADHERENT CELL CULTURE AND ANALYSIS

Chueh-Yu Wu, Daniel Stoecklein, Aditya Kommajosula, Jonathan Lin, Keegan Owsley, Baskar Ganapathysubramanian, and Dino Di Carlo

High-throughput optical Transient Liquid Molding

High-throughput Optical Transient Liquid Molding requires the following components: a microfluidic chip with a pillar design to shape flow, a flow pumping/stopping setup, and a collimated UV light setup. The microfluidic chip was placed on an xy-translational and rotational optical stage as the polymeric precursor liquid (PEGDA with and without photoinitiator) were injected from the bottom of the chip. Particles and unpolymerized waste solution were exhausted out of the bottom of the chip through a pinch valve toward a collection tube. To match the need for increased optical exposure area for our scaled manufacturing, we generated collimated UV light in a 3 cm diameter circle on the top of the chip using a high power UV light source and collimation adaptor (Excelitas Technologies, OmniCure ® S2000 UV Curing System and Adjustable Spot Collimating Adaptor). Instead of projecting one UV light pattern onto the sculpted flow using a mask in the conjugate image plane of the sample (as in the previous version of fabrication [13]), we illuminated collimated UV light through a chrome optical mask, which was placed on the top of the glass substrate of the chip with hard contact. Parallel mask patterns in a linear array were aligned with the channel sculpted flow. We designed alignment marks on the mask and microfluidic chip so the masked patterns could be registered to the same lateral position of the sculpted flow from the first to the last pattern. To verify the collimation, we synthesized 2D extruded microparticles using stationary monomer with photoinitiator inside a microchannel. The angle of the vertical walls of the microparticles was observed to be within ~10 degrees, confirming the collimation. The fluidic and optical setup were controlled with LabVIEW to automate the fabrication process with the following sequential steps: (i) pumping the co-flow using a syringe pump, (ii) stopping flows by stopping the pump and pinching the tubing with a pinch valve downstream of the outlet, (iii) illuminating the flow by opening a shutter mounted below the collimation adaptor, (iv) pumping the solutions again to wash out the cured microcarriers into a conical tube and to rebuild the sculpted flows, and then repeating these steps (i-iv) for multiple cycles. After the final cycle of the process (typically 100 cycles, yielding 10,000 microcarriers), the tube was centrifuged at 2500 rpm for 5 minutes to pull microcarriers down to the bottom of the tube. We then removed supernatant and executed a rinse process with three repeats to remove residual precursor using DPBS with 10^{-3}M pluronic. The rinse process included re-suspending microcarriers in bulk solution, centrifuging microcarriers down to sediment, and gently pipetting out the supernatant, being careful to avoid withdrawing microcarriers.

Simulation of convective diffusion in the fabrication system

To simulate the fabrication error caused by diffusion of photoinitiator away from the sculpted shape, we utilized a finite element method model solving the full 3D incompressible Navier-Stokes equations coupled with 3D diffusion equations in COMSOL Multiphysics. The flow model consisted of fully developed flow at the inlet and outlet, in a rectangular microchannel with 1200µm width, 300µm height, and 2000µm length. The concentration of photoinitiator on the left- and right-hand sides were set to 0 and 1, respectively. In order to simulate the entire length, we programmed a script to iterate mapping the outlet concentration to the inlet one and to run the simulation of convective diffusion repeatedly until reaching the channel length of several cm used in practice. We extracted the concentration distribution for different numbers of iterations to evaluate the degree of fabrication error for different channel lengths.

Genetic algorithm framework for flow sculpting device design

We used the software FlowSculpt (www.flowsculpt.org) which employs a custom genetic algorithm written in C++ to rapidly design micropillar sequences and inlet flow patterns for inertial fluid flow sculpting. An overview of FlowSculpt's application of the genetic algorithm for flow sculpting is as follows. First, a population of candidate solutions (chromosomes) is randomly generated, with each solution encoded as an inlet flow pattern and a micropillar sequence of fixed length (containing information for each micropillar's diameter and location). Each chromosome is then evaluated based on an objective function (also known as a fitness function, described in further detail below) and ranked in order of performance. Then, a new generation of chromosomes is produced from this population by (1) preserving high-performance "elite" chromosomes, (2) selecting parent chromosomes to exchange (crossover) genetic material (inlet pattern and pillar sequence design) to form off-spring, and (3) mutating randomly selected chromosomes. The use of elites preserves the running best solution within the population. For selection, we identify parents using the tournament method, which chooses the highest-ranked chromosomes out of a randomly chosen group within the population, thus rewarding good fitness in the creation of new offspring, while still allowing for genetic variation within the gene pool. Pairs of selected parents will randomly exchange components of their chromosomes to form offspring for the next generation. Mutation randomly alters a chromosome's design, and attempts to push solutions out of local optima and encourage discovery. This process of evaluation, followed by selection, crossover, and mutation, will repeat over the course of many generations until termination criteria are met. FlowSculpt's termination criteria include a stall generation limit (number of generations for which the optimal fitness does not improve), and a maximum number of generations. At termination, the most-fit chromosome is selected as the optimal solution. Since the genetic algorithm is a stochastic process, FlowSculpt repeats the algorithm at least 10 times, and allows the user to specify micropillar sequence length and the number of channels in the inlet pattern. For this work, the fitness function generates a flow shape image for each chromosome using a forward model that is fast, which is then compared to a user-designed target fluid flow shape using a product-moment correlation function [18].

However, before calculating the correlation value, we compute the discrete Fourier transform (DFT) of the target and chromosome images. This exploits the "shift" property of the Fourier transform, whereby the translation of an image will be reported as a phase shift in frequency space, while the magnitude of the transforms will remain the same [S1]. By comparing the magnitude of each image's DFT we introduce translation invariance to the measure of fitness, thereby searching for a fluid flow shape without regard to where it is located within the microchannel cross-section.

Analysis of the fabrication quality of the microcarriers

We took images of microcarriers and the fluorescent cell adhesive regions and used a custom Matlab code to automate analysis and determine the fabrication quality. We diluted the microcarriers, spread a droplet of solution with microcarriers on a microscopy slide, and sandwiched the solution with a cover slide to align microcarriers for imaging, and then repeated these steps to obtain multiple images while minimizing the overlap between microcarriers in each microscopic image. With several images of microparticles using a 4X objective, we used a Matlab code with canny algorithm to detect the boundary of each microcarrier and for each enclosed boundary rotate it until the aspect ratio reaches a maximum value to allow all boundaries to be aligned in the vertical orientation. We overlapped all the detected boundaries using the same mass center and calculated an averaged deviation of the boundaries to qualitatively and quantitatively describe the fabrication quality. The averaged deviation of the boundaries is defined as the average, over 360 degrees, of the deviation of the distance between the single point on the boundary and the overlapped mass center at an angle of all detected boundaries. Moreover, we took bright-field and florescent images on the microcarriers, which were incubated with fluorescent streptavidin. We rotated and overlapped the fluorescent images with respect to the angle and mass center we processed for the bright-field images of the microcarriers. To represent the fabrication quality of the cell adhesive region, we calculated the average, over all microcarriers, of the 2D correlation between each fluorescent image and the averaged one.

Shear stress distribution

We performed finite-volume simulations to analyse shear stresses on our 3D particles in the microchannel flow, with a focus on the shelter zone, to confirm and gauge viability for adherent cells. The 3D flow patterns of the shaped microcarrier in the middle of the channel were simulated by solving for X-velocity of the particle to yield zero drag. The shear-magnitude was computed from the velocity flow field. Fig. S1 shows the magnitude of shear stresses acting on a single microcarrier. We observe that there is a sharp distinction between regions of high, and low shear on the surface, and it is evident that the nook is a region of minimum shear, hence acting as an ideal shelter for cell adhesion in flow. Although the top and bottom XY-surfaces outside the nook exhibit high-shear, the lowest shear-magnitude in the nook is found to be around 0.45 Pa, which is low enough to avoid any physiological

impact on cell-growth [S2]. Additionally, we identify a secondary region of low shear towards the waist of the particle which lies in between high-shear corners near the head- and tail-lobes, and this supports the possibility of cell adhesion on microcarrier side-walls, as observed in the pipetting experiments, shown in Fig. 4(a).

We further examine the shear stress distribution in the shelter at different YZ-planes (Fig. S2 (b)) vs. Y-coordinate, and it is found that shear-magnitude is minimum on YZ-planes which are at the extremities, i.e., at X = 20 and 110 µm, as opposed to planes at the centre where it is maximum. It is apparent that the steep slope of the profile becomes responsible for a sharp difference in velocity gradients between the top z-surfaces of the microcarrier, and the shelter basin. This is consequently reflected in the separation between maximum and minimum profiles for shear-magnitude. Additionally, we note that shear along the peripheral faces on the plane, Z = 0, (Fig. S2 (c)) is non-uniform owing to shape asymmetry, and shear-peaks are evident around sharp bends or corners on the microcarrier.

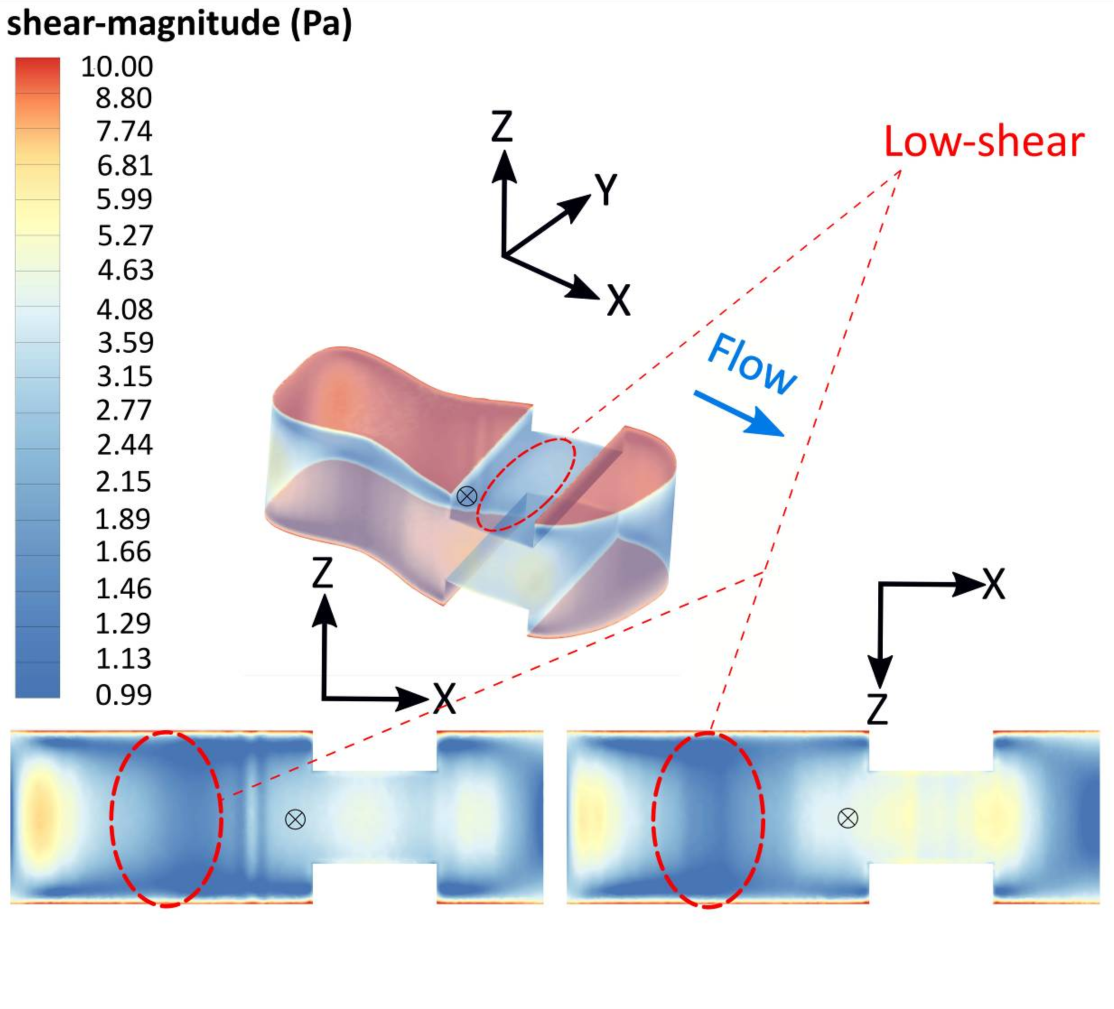

Figure S1. Shear-magnitude distribution (origin at particle centroid, as marked)

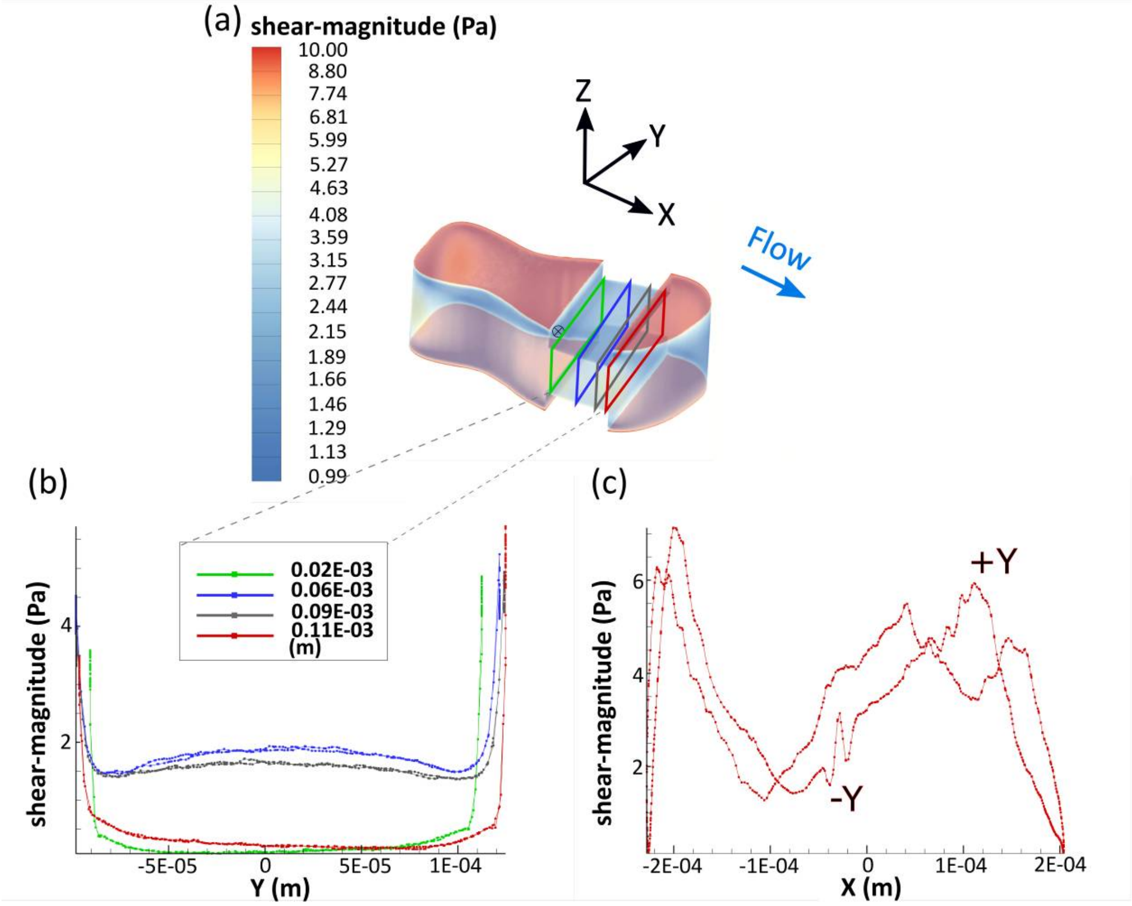

Figure S2. Shear-magnitude distribution (a) on particle, (b) shelter, (c) peripheral surfaces (origin at the centroid of the microcarrier, as marked)

Cell adhesion, proliferation, protection, and analysis

We provide more data demonstrating cell adhesion, proliferation, viability and analysis using 3D microcarriers in Fig. S3 and S4.

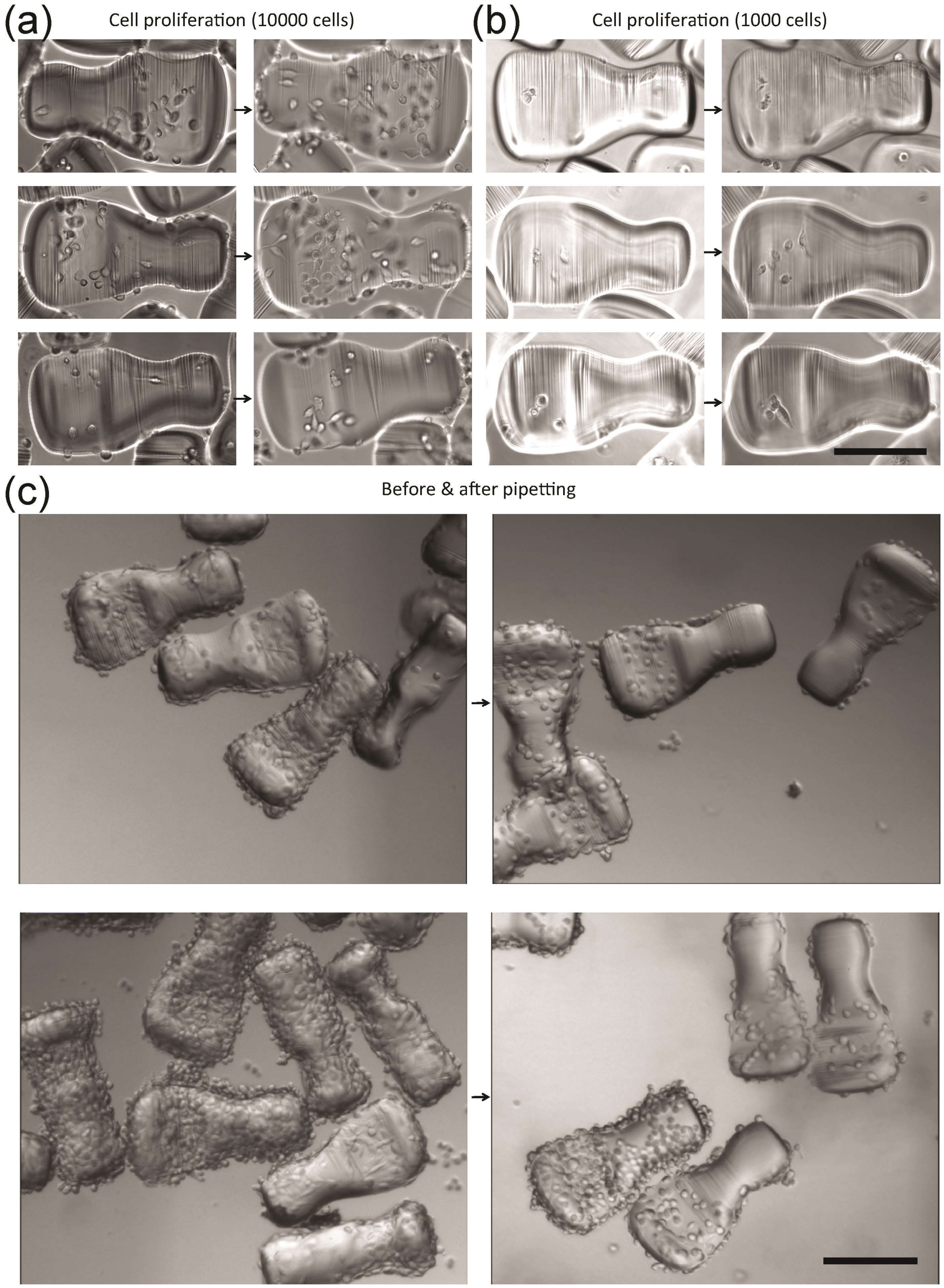

Figure S3. Cell proliferation and protection using 3D microcarriers. Cells attached and grew in the shelter region in data from 250-300 microcarriers and a total cell number of (a) 10,000 and (b) 1,000 cells seeded. (c) Cells were protected in the shelter region during pipetting. Both scale bars are 200 μm.

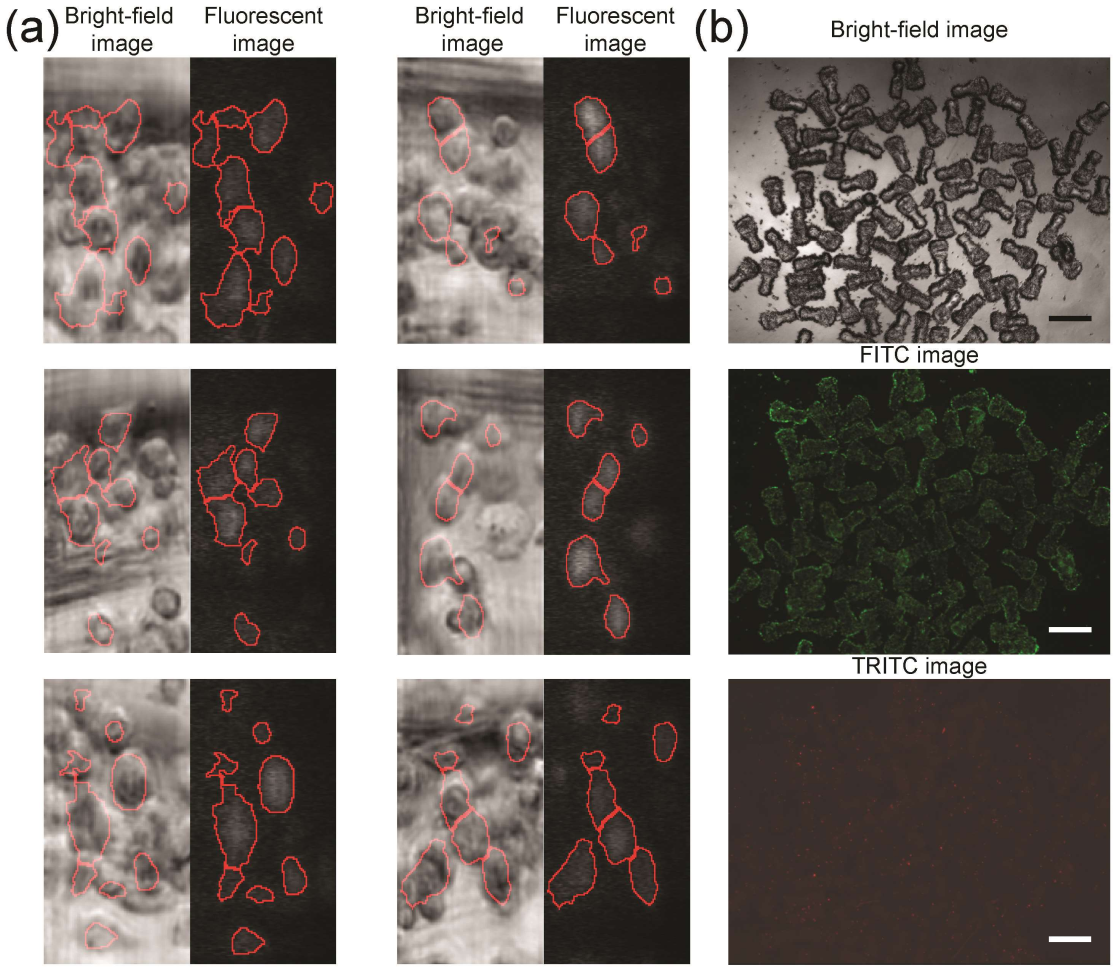

Figure S4. Cell analysis using (a) 3D microcarriers with the FIRE imaging system and (b) an image of viable cells after the flow-through experiment with a 2X objective showing cells were protected in a reproducible fashion across microcarriers. The scale bars on the right hand side are 500 μm.

[S1] B. Srinivasa Reddy and B. N. Chatterji, “An FFT-based technique for translation, rotation, and scale-invariant image registration,” *IEEE Trans. Image Process.*, 5, 8, 1266–1271, 1996.

[S2] X. Cui, W. Guo, Y. Sun, B. Sun, S. Hu, D. Sun, and R. H. W. Lam, "A microfluidic device for isolation and characterization of transendothelial migrating cancer cells." *Biomicrofluidics*,11, 014105, 2017.
